# Structural and Electrochemical Investigation during the First Charging Cycles of Silicon Microwire Array Anodes for High Capacity Lithium Ion Batteries

**DOI:** 10.3390/ma6020626

**Published:** 2013-02-22

**Authors:** Enrique Quiroga-González, Jürgen Carstensen, Helmut Föll

**Affiliations:** Institute for Materials Science, Christian-Albrechts-University of Kiel, Kaiserstr. 2, Kiel 24143, Germany; E-Mails: equiroga@ieee.org (E.Q.-G.); jc@tf.uni-kiel.de (J.C.)

**Keywords:** Li-ion batteries, Si microwire array, Si anode, *in situ* impedance spectroscopy, high capacity, solid electrolyte interface (SEI) formation, Si-Li phases

## Abstract

Silicon microwire arrays embedded in Cu present exceptional performance as anode material in Li ion batteries. The processes occurring during the first charging cycles of batteries with this anode are essential for good performance. This paper sheds light on the electrochemical and structural properties of the anodes during the first charging cycles. Scanning Electron Microscopy, X-ray diffractommetry, and fast Fourier transformation impedance spectroscopy are used for the characterization. It was found that crystalline phases with high Li content are obtained after the first lithiation cycle, while for the second lithiation just crystalline phases with less Li are observable, indicating that the lithiated wires become amorphous upon cycling. The formation of a solid electrolyte interface of around 250 nm during the first lithiation cycle is evidenced, and is considered a necessary component for the good cycling performance of the wires. Analog to voltammetric techniques, impedance spectroscopy is confirmed as a powerful tool to identify the formation of the different Si-Li phases.

## 1. Introduction

Nano- and micro-structured Si anodes have gained much attention in the battery community in the last years because they could allow for very high capacity for Li storage up to the theoretical maximum storage in Si of 4200 mAh/g, as evidenced by some reports [1,2,3,4], which is more than 10 times larger than the capacity of graphite, the most common anode in Li ion batteries. Additionally, nano- and micro-structured Si overcome the problems of pulverization of bulk Si caused by volume expansion during lithiation. These anodes present facile strain relaxation, especially in a wire-like form [2].

Most of the articles dealing with Si anodes report about Si nanowires; originally a maximum thickness of 300 nm of the wires was considered the upper limit for a good performance [5], but in a new report it has been proved that Si microwire array anodes with wires of around 1 µm in diameter present extraordinary charging cyclability, reaching a capacity of 4200 mAh/g, which remained stable for several tenths of cycles [4]. The areal capacity of these anodes is relatively high (6.3 mAh/cm^2^), due to the length (70 µm) and the thickness of the wires. Up to now, not much is known about the exact reasons leading to the extraordinary good performance of these anodes.

Quite obviously the first lithiation/delithiation cycles play an essential role for the following cycling performance of the Si anodes, since in these first cycles the solid electrolyte interface (SEI) is formed, and the crystallization of different Si-Li phases is defined. With respect to the type of Si-Li phases there is much controversy in the scientific community. For example, some groups state that the highest lithiated crystalline Si-Li phase is Li_22_Si_5_ [1,6], exhibiting a potential of 35 mV* vs.* Li/Li^+^; on the other hand, other reports indicate that the maximum lithiation produces a potential slightly below 50 mV, given by the formation of Li_15_Si_4_ [7]. Additionally, the potential of the anodes seems to be also influenced by the size of the Si particles/wires, as observed in [8,9], where the minimum potential of the anodes exceeds 70 mV using larger Si pieces.

In this paper the structural characteristics of Si microwire array anodes in their lithiated state during the first charging cycles are discussed. *In situ* fast Fourier transformation impedance spectroscopy (FFT-IS) is applied for the electrochemical characterization of the anodes during the first lithiation/delithiation cycles. The modeling of the impedance data as well as the interpretation is discussed.

## 2. Results and Discussion

### 2.1. Battery Cycling Test

**Figure 1:**
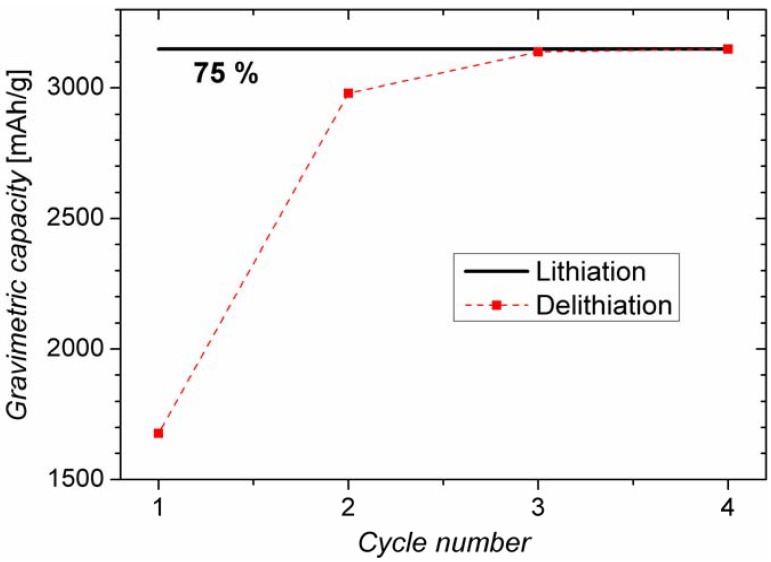
Cycling performance of the Si wire anode with *C*/10 current for four cycles. The battery was charged to 75% of its maximal capacity to reach long term stability.

From previous work it is known that the present anodes can be charged/discharged for 100 cycles without any significant fading (fading close to zero) when charging/discharging to 75% of the maximum capacity of Si and limiting the operation voltage range between 0.11 and 0.7 V [4]. Going beyond these voltage limits (especially in the first two cycles) the cycling performance of the battery drastically decreases. In this paper we only discuss “good” battery behavior. Figure 1 shows the results of a recent measurement, charging/discharging for four cycles, under the same conditions. It is evident that some lithium cannot be extracted from the anode during the first three cycles, but these irreversible losses (around 50%) only show up in these first cycles. They are most probably correlated with the formation of a solid electrolyte interface (SEI), as discussed in the next sections. Additionally, an irreversible process occurring just in the first cycle of cyclic voltammetry on the anodes at around 1.2 V [4] supports this assumption. It has been reported that SEI forms when the voltage is reduced to under 1.3 V [10], the electrochemical stability voltage of an organic electrolyte.

The losses by SEI formation are in the present case not a negative aspect of the anodes, since it is considered a required and essential part of a battery for good performance [11]; thus the lithium incorporated in the SEI is not a loss, but an important component of this essential layer.

### 2.2. Structural Characterization

#### 2.2.1. Unlithiated Anodes

**Figure 2:**
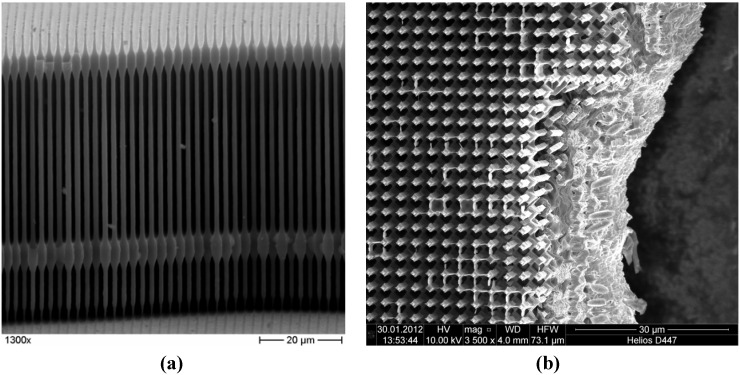
(**a**) Silicon microwire array of the anodes. The wires are 70 µm long and have 1 µm of diameter. The wider parts are stabilizing planes; (**b**) Top view of a finished anode. The bottom 10 µm of the wires are embedded in Cu (visible at the right).

Scanning electron microscope (SEM) micrographs of the Si wire array anode in its unlithiated state are shown in Figure 2. The wires are 70 µm long and have a diameter of 1 µm (see Figure 2a). The wide wire sections are stabilizing planes. In the finished anodes, around 10 µm of the wires are embedded in Cu, allowing for a good electrical contact and leading to a mechanically stable electrode. Figure 2b shows a top view of the Cu contacted anodes.

#### 2.2.2. Lithiated Anodes

**Figure 3:**
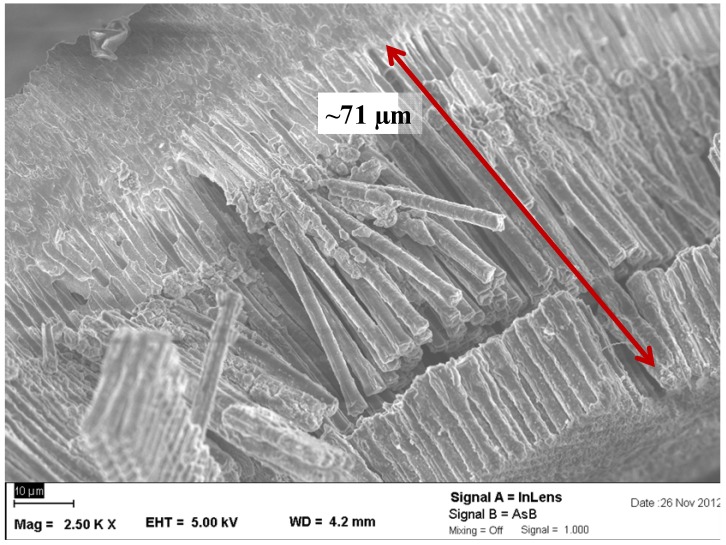
Scanning electron microscope** (**SEM) micrograph of the anodes after the second lithiation cycle. The wires were broken during the preparation of the sample for SEM (a cut in cross section), as confirmed with other micrographs, where the wires behind the cut fronts were complete (see Figure S1). The length of the wires is nearly the same as in the not lithiated state.

A SEM micrograph of an anode after the second lithiation cycle is shown in Figure 3. The length of the wires is around 71 µm, almost the same as the length of the unlithiated wires (70 µm). The small difference of 1.4% is in the range of measurement errors. For these microwires, the necessary volume expansion of Si when lithiated is obviously only significant in the *x* and *y* directions, because the expansion in the *z* direction is negligibly small.

**Figure 4:**
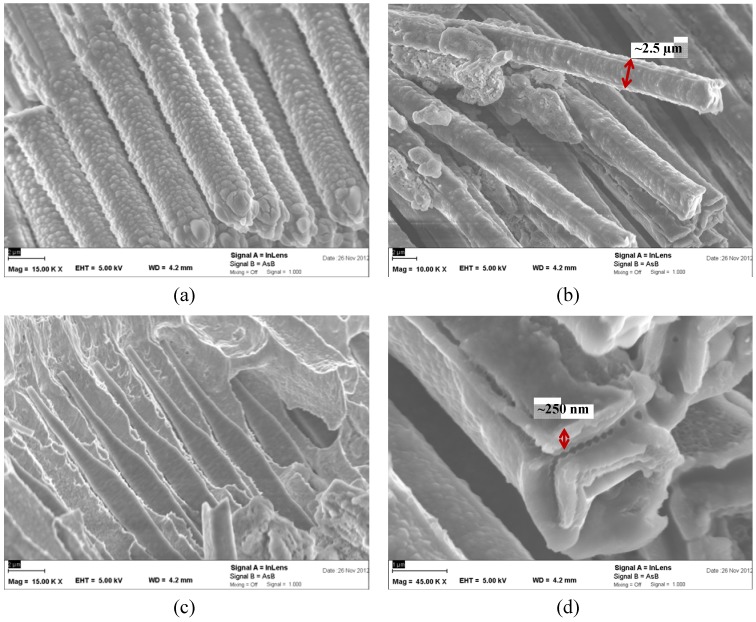
Micrographs of different regions of the same lithiated microwires as shown in Figure 3. (**a**) Tips of the wires; (**b**) Middle section; (**c**) Section embedded in Cu; (**d**) Surface of the wires in the middle section; here the solid electrolyte interface (SEI) is visible.

Micrographs of different sections of the lithiated anodes are shown in Figure 4: Images of the wire tips, of their middle section, of the section embedded in Cu, and of a zoom on the wire surface are shown in Figure 4a–d, respectively. The pristine wires have a quadratic cross-section, but as can be seen from Figure 4a, they become nearly cylindrical upon lithiation. The diameter of the wires at the top and at the middle section are nearly the same, around 2.5 µm, which means an area increase of 214% compared to the pristine wires. In this way, again one can conclude that the main volume expansion of the wires during lithiation takes place diametrically in the *xy* plane in the wire sections not embedded in Cu. The wire sections in Cu remain unaltered (see Figure 4c). The explanation for this is that the size of the wires is constraint by the Cu matrix and no significant volume expansion is possible; diffusion of Li into this region is not forbidden but the concentration of Li in this region will stay low, since the formation of Si-Li phases requires additional space and is thus not possible.

The morphology of the lithiated wire sections varies. In the middle section the wires are smoother than close to the tip. Probably the roughness can be associated with the crystallization of some Li-Si phases inside the wires or of SEI components at the interface, so this difference in roughness is a hint that the lithiation does not take place homogeneously through the wires.

The surface of the wires zoomed in Figure 4d exhibits a thin covering layer of around 250 nm. This layer is most probably the SEI that of course covers the whole wires. This observation is representative for the present wires, where many SEM images were recorded. 

**Figure 5:**
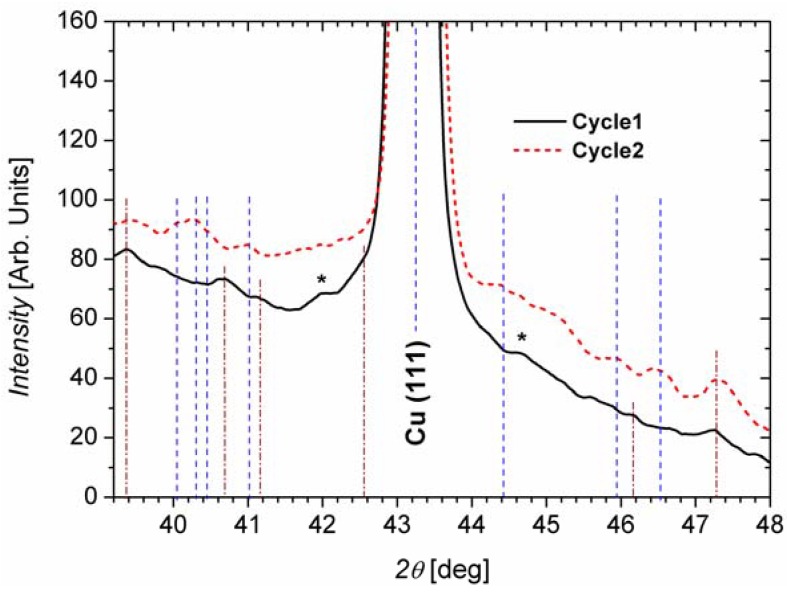
X-ray diffractograms of lithiated Si wire anodes after one and two charging cycles. The vertical lines are a guide for the eye to identify the different peaks. The peaks marked with an asterisk could not be assigned to a single Si-Li phase; in those ranges many low intensity reflections of different Li-Si phases are possible.

X-Ray Diffraction (XRD) patterns of Si anodes lithiated in one and two charging cycles are shown in Figure 5. The diffractograms were recorded in the *2θ* range where the most intense reflexes of Li-Si phases are expected (39°–48°). The most prominent peak is found at 43.26°, corresponding to Cu (111) [12]. The vertical lines in the figure are a guide for the eye to indicate where some peaks related to Si compounds are identified. Making a survey among all the possible Li-Si phases, the best match was done taking into account the intensity and the position of the reflexes. The position and possible origin of the different identified XRD peaks are shown in Table 1. The regions marked with asterisks in Figure 5 do not allow for a clear identification of Li-Si phases since many low intensity reflexes of several phases are possible here.

As can be seen in Table 1, crystalline Si-Li phases with high Li content are found after the first lithiation cycle (Li_22_Si_5_ [13] and Li_13_Si_4_ [14]), while mainly phases with lower Li content are found after the second lithiation (Li_12_Si_7_ [1] and Li_2_Si [15]). The crystalline nature of the pristine wires may enable the crystallization of high lithiated phases, while for the following cycles, when the wires start getting amorphous, just crystal structures with fewer atoms are possible. This does not mean that the amount of Li which can be incorporated into Si reduces after the first two cycles; it just means that the fraction of crystalline areas reduces in favor of amorphous areas, which cannot be analyzed using XRD. Since no fading of the electrodes is found for all subsequent cycles, there is no experimental hint that the amount of Li which can be stored in the Si matrix reduces by cycling.

Besides the Si-Li phases, also Si [16] and Li_5_SiF_6_ [17] have been identified. The presence of Si reflexes is in accordance with the Si wire sections embedded in Cu, where no Si-Li phases can be formed. On the other hand, there is also an indication that Li_5_SiF_6_ could be present in the SEI. The SEI in Li ion batteries usually consists of decomposition compounds of the battery electrolyte, of organic and inorganic compounds of the solvents, and of the anions of the LiPF_6_ salt [10].

**Table 1:** X-Ray Diffraction (XRD) peaks from lithiated Si microwire anodes after the first and second charging cycles.

Cycle 1			Cycle 2		
Measured 2*θ* [deg]	Literature 2*θ* [deg]	Origin (hkl)	Measured 2*θ* [deg]	Literature 2*θ* [deg]	Origin (hkl)
39.4	39.5	Li_5_SiF_6_ (002)	39.44	39.5	Li_5_SiF_6_ (002)
40.72	40.81, 40.71	Li_22_Si_5_ (660), Li_13_Si_4_ (002)	39.97	39.88	Li_12_Si_7_ (254)
41.16	41.27	Li_13_Si_4_ (321)	40.28	40.22	Li_12_Si_7_ (272)
42.5	42.55	Li_13_Si_4_ (251)	40.5	40.64	Li_2_Si (−311)
46.16	46.13	Li_13_Si_4_ (132)	40.96	40.9	Li_2_Si (020)
47.24	47.06	Si (220)	44.44	44.54	Li_12_Si_7_ (084)
–	–	–	45.9	45.9	Li_12_Si_7_ (010 0)
–	–	–	46.45	46.39, 46.6	Li_12_Si_7_ (037), Li_2_Si (−113)
–	–	–	47.29	47.06	Si (220)

### 2.3. *In Situ* Impedance Spectroscopy

Impedance spectroscopy (IS) is a powerful method able to produce relevant information for electrochemical processes and has been used to analyze Si anodes, mostly post-mortem or *ex situ* [18,19]. Two main time constants have been identified, a fast one related to interfacial characteristics, and a slow one related to Li diffusion in the electrodes [18,19]. The solid state diffusion of Li in Si could be understood using a Warburg impedance for modeling the impedance data in a frequency well below 1 Hz. Not much is known about the evolution of the impedance parameters during the lithiation and delithiation processes or about faster processes. Therefore in this paper FFT-IS results will be discussed where 30 frequencies in the range from 5 Hz to 20 kHz are applied simultaneously to current or voltage as a perturbation.

**Figure 6:**
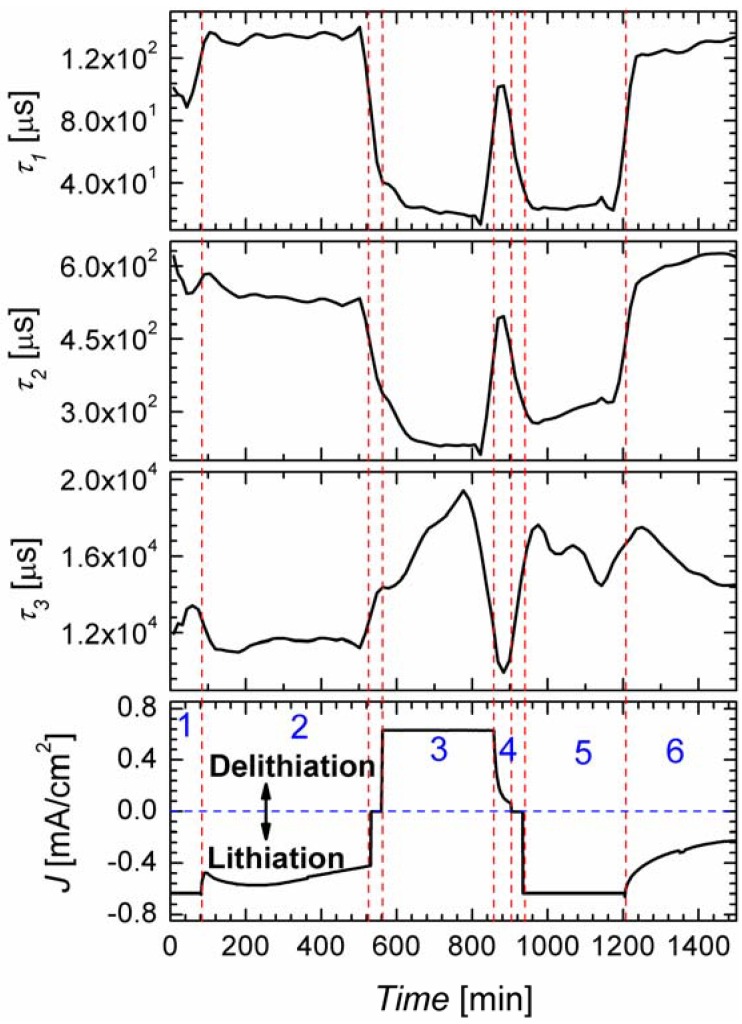
Plots of the different time constants obtained by fast Fourier transformation impedance spectroscopy (FFT-IS) during the lithiation/delithiation process of the wires. The current density *J* during cycling is shown as reference. The plots are divided into six sections. Sections 1, 3 and 5 are obtained under constant current, and Sections 2, 4 and 6 under constant voltage.

The impedance data was fitted with a model of three *RC* circuits (producing the time constants *τ*_1_, *τ*_2_, and *τ*_3_, and the resistances *R*_p1_, *R*_p2_, and *R*_p3_) and a resistor in series *R*_s_. Plots of the time constants for the first two lithiation cycles are shown in Figure 6. The order of magnitude for the time constants as well as the values for the resistances for the processes 2 and 3 correspond well the two processes found in IS under open circuit condition [20]. Without going into details the time constants can most probably be related to ionic conduction through the electrolyte (*τ*_1_, fastest process), charge transfer through the SEI (*τ*_2_), and charge transfer to the Si microwires (*τ*_3_, slowest process). Figure 6 reveals that the first two time constants *τ*_1_ and *τ*_2_ show a similar behavior upon cycling. The variation of the time constants in the different phases of the cycles is roughly one order of magnitude. Charging/discharging in galvanostatic mode,* i.e.*, applying a constant current is accompanied by nearly constant small values of the time constants *τ*_1_ and *τ*_2_. In the phases of constant potential the time constants *τ*_1_ and *τ*_2_ are much larger. This is probably not related to potentiostatic and galvanostatic control of the experiment but to the state of battery charging. The voltage limits (at which the charging/discharging is switched to potentiostatic mode) mark the nearly fully lithiated state or the nearly fully delithiated state of the Si microwires; due to this, when the constant voltage is activated, the charging/discharging may become more difficult and thus it is a slow process.

**Figure 7:**
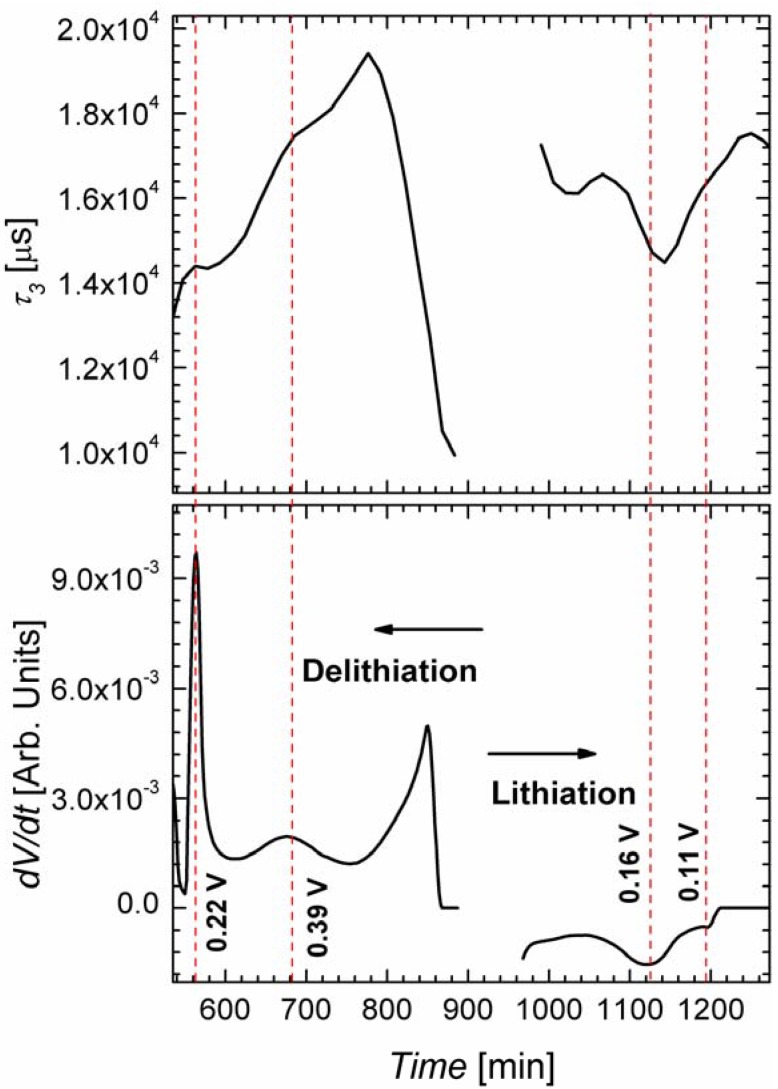
Plot of the time constant *τ*_3_ with time in the range of constant current. The derivative of the voltage in those ranges is also shown.

Time constant *τ*_3_ presents a different behavior; it varies much less in magnitude upon cycling. Mainly during the galvanostatic mode some small changes of the time constant *τ*_3_ are observable. This could be explained by the successive formation of different Si-Li phases, which coincide with the corresponding steps in the measured potential (not shown here). In the potentiostatic phase the potential is fixed to its highest (for lithiation) or lowest (for delithiation) value, only allowing for one Si-Li phase to be formed and thus for a nearly constant *τ*_3_. In Figure 7 the time constant *τ*_3_ is shown for the galvanostatic mode; in addition the derivative of the anodic potential is plotted. The maxima/minima of this curve represent the inflection points of the voltage; the standard interpretation of such inflection points is that here the formation of a new Si-Li phase starts. The curve of *τ*_3_ kinks exactly at these points, which is a strong indication that the time constant *τ*_3_ is related to the charge transfer to the Si wires. This interesting observation proves the power of the FFT-IS technique for characterizing Li ion batteries.

## 3. Experimental Section

Silicon microwire anodes have been produced by an electrochemical-chemical approach thoroughly described in [3,9]. The steps for the preparation of the Si wire anodes could be summarized as follows: (a) Cheap lithography and pre-structuring of Si wafers; (b) electrochemical etching of macropores; (c) chemical over-etching of macroporous Si to obtain a wire array; (d) chemical-electrochemical deposition of a Cu current collector on the wires; (e) detaching the anode from the (reusable) Si substrate.

Lithiation/delithiation tests were performed using the following conditions: A current of *C*/10 (the current is calculated so that it takes 10 h to lithiate or delithiate) was used. For the lithiation a voltage limit of 0.11 V was taken, while for the delithiation the voltage limit was 0.7 V,* i.e.*, when these voltage limits are reached, the cycling is switched to potentiostatic mode, and this mode finished when the current decreased to 10%, or the capacity limit of 3150 mAh/g (75% of the theoretical maximum) is reached. This is a standard procedure for charging commercial batteries to protect them from over-charging. The wires were charged to 75% of the maximal Li content to avoid unnecessary stresses in the wires which occur especially when totally dealloying the Si from Li [4].

FFT-IS measurements were performed *in situ* during lithiation/delithiation by applying 30 frequencies in a range of 5 Hz to 20 kHz simultaneously to the current. The FFT-IS technique allows us to analyze the linear response of all frequencies in parallel. An FFT-spectrum was taken every 5 s, and 12 spectra have been averaged in order to reduce the noise level and amount of data. All spectra could be fitted quite well by a simple equivalent circuit consisting of a resistor in series with three circuits of a resistor parallel to a capacitor (*R*_p_*C*_p_ components = time constants *τ*). A potentiostat of ET&TE GmbH (Kiel, Germany) was used for the lithiation/delithiation of the anodes and the impedance analysis.

For the electrochemical characterization of the anodes, half-cells were prepared, with Li as reference electrode. The separator was a glass fiber filter from Whatman, with pores of 1 µm. The electrolyte was LP-30, consisting of dimethylcarbonate and ethylencarbonate (1:1) plus 1 mol/L of LiPF_6_.

The structural characterization of the anodes was performed by means of XRD and SEM. A Seifert XRD 3000 TT tool was used for recording diffraction patterns of the anodes in reflection mode using a Cu Kα beam with a wavelength of 0.154 nm. Lithiated samples were taken out of the battery case and were covered with scotch tape inside an Ar-filled glove box for the XRD measurements. Amorphous silica glass was used as support. SEM investigations were performed with an Ultra Plus SEM from ZEISS.

## 4. Conclusions

Si microwire anodes present an extraordinary charging performance especially with respect to the high Li storage capacities. SEM micrographs, XRD curves, and *in situ* FFT-IS have been used to characterize the Si microwire anodes, when cycling under optimal conditions,* i.e.*, limiting the applied voltages between 0.11 V and 0.7 V. The formation of an SEI has been verified by the SEM and FFT-IS analysis. Several Si-Li phases could be identified in different charging states of the wires by XRD, cyclovoltammetry, and FFT-IS. In future work these tools will be used to characterize the mechanisms of destructing the electrode, e.g., when charging with higher voltages than 0.7 V. This knowledge may help to improve the battery stability further and/or allow for higher charging currents.

## Supplementary materials

Supplementary File 1
