# Supplementary material for: Structural and Electrochemical Investigation during the First Charging Cycles of Silicon Microwire Array Anodes for High Capacity Lithium Ion Batteries
Source: Materials (Basel). 2013 Feb 22;6(2):626–36. doi: 10.3390/ma6020626 (PMC5452100; doi:10.3390/ma6020626)
Supplement: Supplementary File 1 [file materials-06-00626-s001.pdf]

Supplementary Information

Figure S1. Scanning electron microscope (SEM) micrograph of the anodes after the second lithiation cycle. Some wires were broken during the preparation of the sample for SEM, but the rest behind the cut fronts are complete. The wires remain complete during cycling, as confirmed by the extraordinary capacity retention over 100 cycles (fading close to zero).

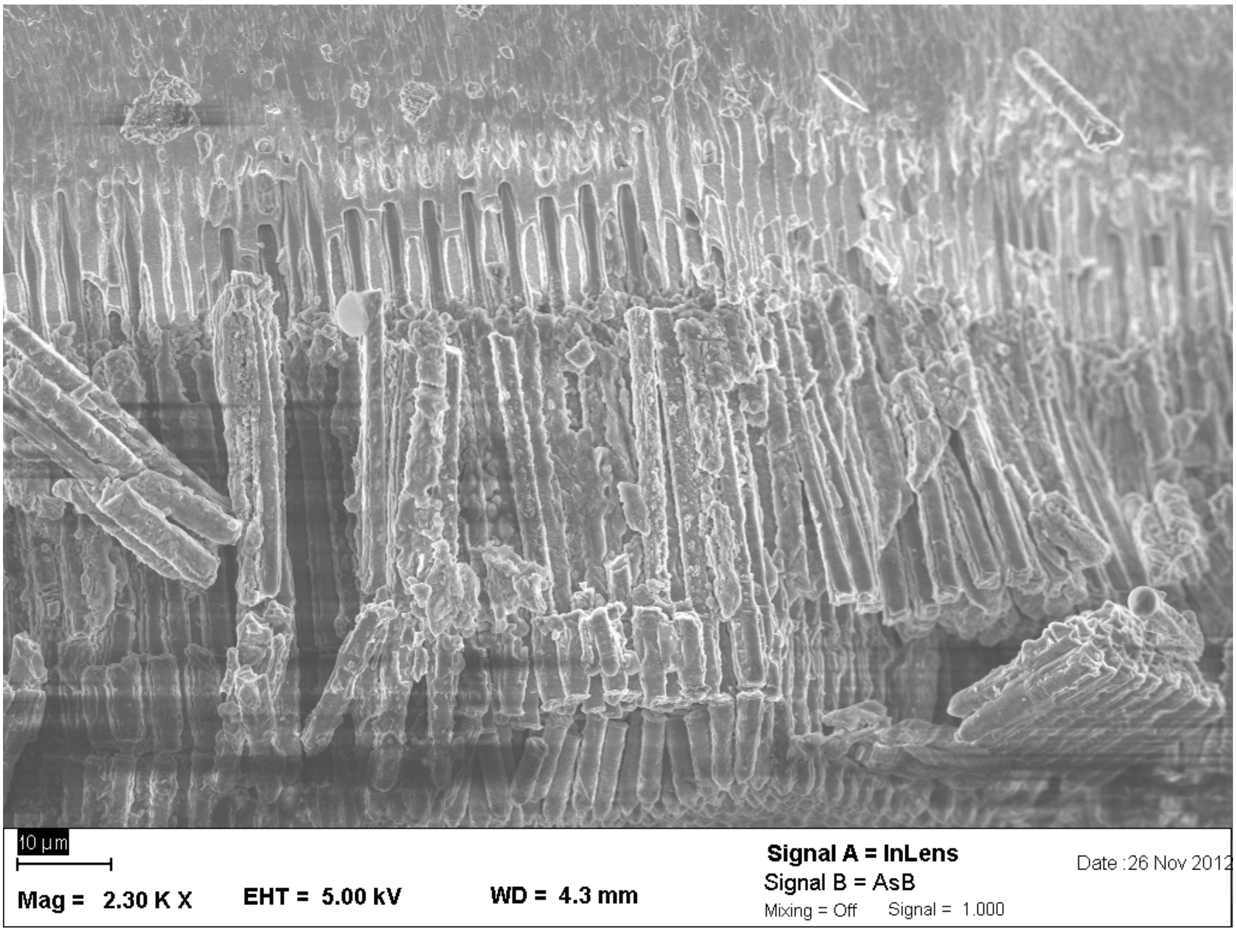

© 2013 by the authors; licensee MDPI, Basel, Switzerland. This article is an open access article distributed under the terms and conditions of the Creative Commons Attribution license (http://creativecommons.org/licenses/by/3.0/).
